# A novel Keap1 inhibitor iKeap1 activates Nrf2 signaling and ameliorates hydrogen peroxide-induced oxidative injury and apoptosis in osteoblasts

**DOI:** 10.1038/s41419-021-03962-8

**Published:** 2021-07-05

**Authors:** Yue-huan Zheng, Jian-jun Yang, Pei-jun Tang, Yuan Zhu, Zhe Chen, Chang She, Gang Chen, Peng Cao, Xiang-yang Xu

**Affiliations:** 1 Department of Orthopedics, Ruijin Hospital, Shanghai Jiao Tong University School of Medicine, Shanghai, China; 2 Department of Orthopedics, Tenth People’s Hospital of Tongji University, Shanghai, China; 3 Department of Pulmonary, The Affiliated Infectious Diseases Hospital of Soochow University, The Fifth People’s Hospital of Suzhou, Suzhou, China; 4 Department of Orthopedics, The Second Affiliated Hospital of Soochow University, Suzhou, China

**Keywords:** Apoptosis, Stress signalling

## Abstract

An ultra-large structure-based virtual screening has discovered iKeap1 as a direct Keap1 inhibitor that can efficiently activate Nrf2 signaling. We here tested its potential effect against hydrogen peroxide (H_2_O_2_)-induced oxidative injury in osteoblasts. In primary murine and human osteoblasts, iKeap1 robustly activated Nrf2 signaling at micromole concentrations. iKeap1 disrupted Keap1-Nrf2 association, causing Nrf2 protein stabilization, cytosol accumulation and nuclear translocation in murine and human osteoblasts. The anti-oxidant response elements (ARE) activity and transcription of Nrf2-ARE-dependent genes (including *HO1*, *NQO1* and *GCLC*) were increased as well. Significantly, iKeap1 pretreatment largely ameliorated H_2_O_2_-induced reactive oxygen species production, lipid peroxidation and DNA damage as well as cell apoptosis and programmed necrosis in osteoblasts. Moreover, dexamethasone- and nicotine-induced oxidative injury and apoptosis were alleviated by iKeap1. Importantly, Nrf2 shRNA or CRISPR/Cas9-induced Nrf2 knockout completely abolished iKeap1-induced osteoblast cytoprotection against H_2_O_2_. Conversely, CRISPR/Cas9-induced Keap1 knockout induced Nrf2 cascade activation and mimicked iKeap1-induced cytoprotective actions in murine osteoblasts. iKeap1 was ineffective against H_2_O_2_ in the Keap1-knockout murine osteoblasts. Collectively, iKeap1 activated Nrf2 signaling cascade to inhibit H_2_O_2_-induced oxidative injury and death of osteoblasts.

## Introduction

In the pathogenesis of osteoporosis or osteonecrosis, reactive oxygen species (ROS) overproduction will cause oxidative injury to osteoblasts, leading to sustained bone injury [1–3]. In vitro, hydrogen peroxide (H_2_O_2_) was added to cultured osteoblasts/osteoblastic cells to mimic oxidative injury. It is a cellular model of osteoblast oxidative injury [4–10]. H_2_O_2_ stimulation in osteoblasts/osteoblastic cells should induce profound oxidative injury, lipid peroxidation, protein denaturation, and significant DNA damage. These events will together cause death of osteoblasts [5–8, 10].

Nuclear factor E2-related factor 2 (Nrf2) is an endogenous cellular defensive mechanism against various oxidative stimuli [11–13]. The unstimulated Nrf2 protein stays in cytosol and directly binds to Keap1 (Kelch-like ECH-associated protein 1) [14, 15]. Keap1 will promote Nrf2 protein degradation through the ubiquitin-proteasome system by Cullin 3 (Cul3) [13, 16]. Activated Nrf2, however, will separate from Keap1 [11–13]. Stabilized Nrf2 protein then translates to cell nuclei and binds to anti-oxidant response elements (ARE) as well as small MAF transcription factors. It will then lead to transcriptional activation and expression of a large number of Nrf2-dependent genes [11–13]. The majority of Nrf2-dependent genes are anti-oxidant genes and detoxifying enzymes, including *heme oxygenase 1* (*HO1*), *NAD(P)H quinone oxidoreductase-1* (*NQO1*), *glutathione (GSH)*, *γ-glutamyl cysteine ligase catalytic subunit* (*GCLC*), and *modified subunit* (*GCLM*), among others [11–13].

Studies have shown that activation of Nrf2 cascade could protect osteoblasts/osteoblastic cells from H_2_O_2_. MIND4-17 can uniquely activate Nrf2 cascade by directly disrupting Keap1-Nrf2 association. Guo et al. found that MIND4-17 activated Nrf2 signaling in primary osteoblasts to ameliorate H_2_O_2_-induced oxidative injury and apoptosis [10]. Han et al. reported that chlorogenic acid protected MC3T3-E1 osteoblastic cells against H_2_O_2_-induced oxidative damage via activation of PI3K/Akt-dependent Nrf2 pathway [9]. Cul3 is the ubiquitin E3 ligase for Nrf2 protein degradation [17, 18]. Xu et al. reported that Cul3 silencing by microRNA-455 induced Nrf2 protein stabilization and Nrf2 signaling activation, thereby protecting human osteoblasts/osteoblastic cells from H_2_O_2_ [7]. Four-octyl itaconate (4-OI), a cell-permeable itaconate derivative, is able to activate Nrf2 signaling through alkylating cysteine residues of Keap1 [19–22]. Our previous study has shown that 4-OI activated Nrf2 cascade and inhibited H_2_O_2_-induced oxidative injury in osteoblasts [4].

Thus, Nrf2 signaling activation, using genetic methods or pharmacological strategies, can efficiently protect osteoblasts/osteoblastic cells from H_2_O_2_-induced oxidative injury [4, 7, 9, 10, 23]. A recent study by Gorgulla et al. has carried out an ultra-large structure-based virtual screening on computer clusters and identified a novel Keap1 inhibitor, iKeap1. It can bind to Keap1 with high affinity and block Keap1-Nrf2 association at submicromolar concentrations [24]. In the present study, we found that this novel Keap1 inhibitor activated Nrf2 signaling to inhibit H_2_O_2_-induced oxidative injury and death of osteoblasts.

## Materials and methods

### Reagents, chemicals, and antibodies

iKeap1 was synthesized by Ruilu Chemicals (Shanghai, China) based on its structure [24]. Dexamethasone (Dex), nicotine, and H_2_O_2_ were provided by Sigma Aldrich Chemicals (St Louis, Mo). Antibodies for HO1 (#70081), NQO1 (#3187), Nrf2 (#12721), Keap1 (#8047), Tubulin (#2125), and Lamin B1 (#13435) as well as cleaved-caspase-3 (#9664), cleaved-poly (ADP-ribose) polymerase (PARP, #5625) and cleaved-caspase-9 (#20750) were provided by Cell Signaling Tech China (Shanghai, China). The anti-GCLC antibody (ab55435) and the anti-adenine nucleotide translocase-1 (ANT1) antibody (ab102032) were purchased from Abcam China (Shanghai, China). The anti-cyclophilin-D (CyPD, sc-137136) antibody was purchased from Santa Cruz Biotech (Santa Cruz, CA). From Thermo Fisher Invitrogen (Suzhou, China) RNA assay reagents were obtained. Cell Counting Kit-8 (CCK-8) was purchased from Dojindo Laboratories (Kumamoto, Japan). All cell culture reagents, including fetal bovine serum, DMEM, and antibiotics, were provided by Gibco (Shanghai, China).

### Culture of primary murine and human osteoblasts

The trabecular bone fragments of healthy donors (undergoing pre-implant bony reconstruction of the mandible) were minced into small pieces, washed with cold PBS, and then digested with 2 mg/mL collagenase type II (300 U/mg; Sigma) for 2 h. As reported [25], the primary human osteoblasts were then placed in culture flasks and cultured in DMEM nutrient mixture F-12 (DMEM/F12) supplemented with 10% Fetal Clone I (Hyclone; Thermo Fisher Scientific) and antibiotics, and incubated in a humidified air with 5% CO_2_ at 37 °C. The medium was changed twice a week until cells reached confluence. Written informed consent was obtained from each donor. The culture of primary murine osteoblasts was described in our previous study [4]. Primary osteoblasts were utilized at passage 3–10. The primary osteoblasts were subjected to mycoplasma and microbial contamination examination. STR profiling, population doubling time, and morphology were checked to confirm their genotypes. The protocols of using primary osteoblasts were approved by Ethics Board of Shanghai Ruijin Hospital, in according with the Declaration of Helsinki.

### Quantitative real-time PCR (qRT-PCR)

Murine or human osteoblasts were seeded into six-well plates at 1.2 × 10^5^ cells per well and were subjected to applied treatments. As reported previously [4], total cellular RNA was extracted using TRIzol reagents and was quantified. A SYBR Green PCR kit (Applied Biosystems, Suzhou, China) was employed to perform the qRT-PCR assays under an ABI Prism-7900H Fast Real-Time PCR system [26]. The melting curve analysis was always performed and a 2^−∆∆*C*t^ method was utilized for data quantification. Glyceraldehyde-3-phosphatedehydrogenase(*GAPDH*) was always tested as the internal control and the reference gene. Primers utilized in this study were provided by Dr. Jiang at Nanjing Medical University [26, 27].

### ARE reporter activity

Osteoblasts were seeded into six-well plates at 1.2 × 10^5^ cells per well and transduced with an ARE-inducible firefly luciferase vector (from Dr. Jiang [26] at Nanjing Medical University). After applied treatments, the relative ARE firefly luciferase activity was tested via quantification of the luminescence, and results were always normalized to control.

### Western blotting

Murine or human osteoblasts were seeded into six-well plates at 1.2 × 10^5^ cells per well and were subjected to treatments. Cell lysates were achieved by incubating cells with the cell lysis buffer (Beyotime Biotechnology, Wuxi, China). The nuclei isolation kit (from Sigma, Shanghai, China) was utilized to separate nuclear fraction lysates [28]. Western blotting procedures were described previously [26]. The quantification of the indicated protein bands was through an ImageJ software (from NIH). The same set of lysates were run in parallel gels when necessary.

### Co-immunoprecipitation (Co-IP)

As reported previously [4], following the applied treatments total cell lysates (800 μg proteins per treatment) were pre-cleared and then incubated with an anti-Keap1 antibody (Santa Cruz Biotech, Shanghai, China) overnight. Proteins that were immunoprecipitated with Keap1 were captured by protein IgA/G beads, and were subsequently tested by western blotting. The detailed protocols of isolating mitochondrial fraction lysates and mito-IP were described before [29].

### Cell viability

Murine or human osteoblasts were seeded into 96-well plates (at 3.5 × 10^3^ cells per well) and were subjected to applied treatments. A CCK-8 assay kit was utilized to test cell viability according to the attached protocol. CCK-8 optical density (OD) was tested at 490 nm in each well.

### NQO1 activity assay

The detailed protocols of analyzing NQO1 activity in osteoblasts were described elsewhere [21]. In brief, the inducer potency was quantified by the use of the NQO1 bioassay. Murine or human osteoblasts were seeded into 96-well plates (at 3.5 × 10^3^ cells per well) and were subjected to applied treatments. The NQO1 enzyme activity was quantified in cell lysates using menadione as the substrate. Its value was always normalized to that in control osteoblasts.

### Cell apoptosis-related assays

The detailed protocols of cell apoptosis-related assays, including caspase-3 activity, Annexin V-propidium iodide (PI)-FACS, and nuclear TUNEL (terminal deoxynucleotidyl transferase dUTP nick end labeling) staining assays were described in detail elsewhere [28, 30, 31].

### Mitochondrial depolarization

JC-1 dye (Sigma) will accumulate in mitochondria in cells with mitochondrial depolarization to form monomers and will emit green fluorescence [32]. Murine or human osteoblasts were seeded into six-well plates at 0.8 × 10^5^ cells per well and were subjected to applied treatments. Cells were incubated with JC-1 dye [33]. The JC-1 green fluorescence intensity (at 490 nm) was recorded and the representative JC-1 images were presented.

### ROS detection

The detailed protocols were described in our previous study [4]. Murine or human osteoblasts were seeded into six-well plates at 0.8 × 10^5^ cells per well and were subjected to applied treatments. Osteoblasts were then stained with CellROX (5 μM, Invitrogen-Thermo Fisher) for 30 min at room temperature. A fluorescent spectrophotometer was employed to examine CellROX fluorescence intensity. The representative CellROX images were presented as well.

### Lactate dehydrogenase (LDH) assay of cell necrosis

Murine and human osteoblasts were seeded into 12-well tissue-culture plates at 5–6 × 10^4^ cells per well. Following the applied treatment, medium and total LDH contents were tested by a two-step simple LDH assay kit. Medium LDH contents were normalized to total LDH.

#### Lipid peroxidation

Murine or human osteoblasts were seeded into six-well plates at 1.2 × 10^5^ cells per well and were subjected to the applied treatments. A thiobarbituric acid reactive substances (TBAR) activity assay was employed to quantitatively measure cellular lipid peroxidation levels using the described protocols [34, 35]. Its level was always normalized to that of control.

#### Single strand DNA (ssDNA) ELISA

Murine or human osteoblasts were seeded into 96-well plates (at 3.0 × 10^3^ cells per well) and were subjected to applied treatments. ssDNA contents were tested through an ApoStrandTM ELISA kit (BIOMOL International, Plymouth Meeting, PA). The ssDNA ELISA absorbance was tested at 450 nm in each well.

### Nrf2 short hairpin RNA (shRNA)

As reported early [4], the primary murine osteoblasts were seeded into six-well plates at 0.8 × 10^5^ cells per well in polybrene (2.5 μg/mL)-containing complete medium and were treated with the Nrf2 shRNA lentiviral particles (sc-37030V, Santa Cruz Biotech, Santa Cruz, CA). Afterward, osteoblasts were returned back to the complete medium, and puromycin (2.5 μg/mL) was added to select stable osteoblasts (for four passages). Nrf2 knockdown was verified by western blotting and qRT-PCR assays.

### Gene knockout (KO) by CRISPR-Cas9 gene editing

Using the described protocol [4] primary murine osteoblasts were seeded into six-well plates at 0.8 × 10^5^ cells per well in polybrene-containing complete medium and transduced with a CRISPR/Cas9-Nrf2-KO-GFP-puro construct or a CRISPR/Cas9-Keap1-KO-GFP-puro construct (see our previous study [4]). The transduced osteoblasts (with GFP) were subjected to FACS-mediated GFP sorting. Osteoblasts were thereafter distributed into 96-well plates, and single stable cells were subjected to Keap1/Nrf2-KO screening. Western blotting and qRT-PCR assays were employed to verify Nrf2 KO or Keap1 KO in single stable osteoblasts. Control osteoblasts were transduced with an empty CRISPR/Cas9-KO-GFP-puro construct (“Cas9-C”).

### Statistical analysis

Quantitative data, all with normal distribution, were shown as mean ± standard deviation (SD). Statistical analyses between multiple groups were examined using ANOVA plus a Scheffe’s *f*-test (SPSS 23.0, SPSS Co. Chicago, IL). To examine significance between two treatment groups, a two-tailed unpaired *T* test (Excel 2007) was employed. Values of *P* < 0.05 were considered as statistically significant.

## Results

### iKeap1 activates Nrf2 cascade in murine and human osteoblasts

**Fig. 1:**
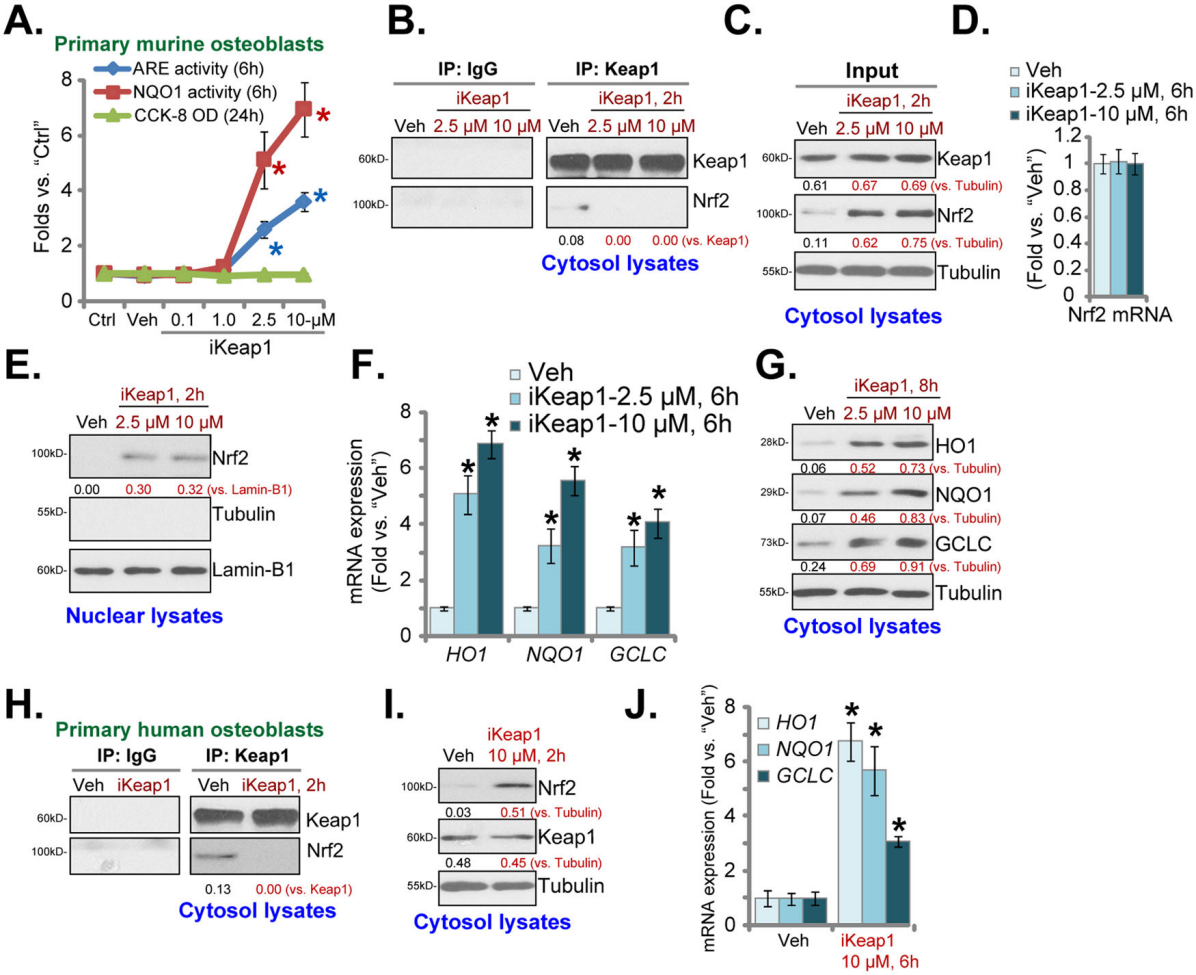
iKeap1 activates Nrf2 cascade in murine and human osteoblasts. The primary murine osteoblasts (**A**–**G**) or the primary human osteoblasts (**H**–**J**) were treated with applied concentration of iKeap1 (0.1–10 μM), cells were further cultured for indicated time points, the relative ARE activity, NQO1 activity, and cell viability (CCK-8 OD) were tested (**A**); Keap1-Nrf2 association was tested by the co-immunoprecipitation (Co-IP) assays (**B**, **H**); Expression of listed proteins in cytosol fraction lysates and nuclear fraction lysates were examined by western blotting assays (**C**, **E**, **G**, **I**), with relative expression of listed mRNAs tested by qRT-PCR assays (**F**, **J**). Expressions of the listed proteins were quantified and normalized to the loading control. Quantified values were mean ± standard deviation (SD, *n* = 5). “C” stands for the untreated control cells. “Veh” stands for the vehicle control (0.1% DMSO). * *P* < 0.05 vs. “Veh” cells. Experiments were repeated five times, with similar results obtained.

First, we examined whether iKeap1 could activate Nrf2 signaling in osteoblasts. The primary murine osteoblasts (see our previous study [4]) were treated with iKeap1 at gradually increased concentrations, from 0.1–10 μM, and cultured for 6 h. Cellular ARE activity was tested and results showed that iKeap1 dose-dependently increased ARE activity in murine osteoblasts (Fig. 1A). ARE activity increase was significant after 2.5–10 μM of iKeap1 treatment (*P* < 0.05 vs. vehicle control, Fig. 1A). In addition, iKeap1 (at 2.5–10 μM) potently increased NQO1 activity (Fig. 1A). These results implied that iKeap1 dose-dependently activated Nrf2 cascade in murine osteoblasts (Fig. 1A). In contrast, iKeap1, at the tested concentrations (0.1–10 μM, for 24 h), failed to inhibit viability (CCK-8 OD) of the murine osteoblasts (Fig. 1A). At the two concentrations, 2.5 and 10 μM, iKeap1 efficiently increased activities of ARE and NQO1 (Fig. 1A). These two concentrations were selected for the following studies.

Next, the Co-IP assay was employed, and results demonstrated that iKeap1 (2.5 and 10 μM, 2 h) disrupted Keap1-Nrf2 association in primary murine osteoblasts (Fig. 1B). Consequently, Nrf2 protein was stabilized in the cytosol (Fig. 1C). Keap1 protein expression was unchanged after the applied iKeap1 treatment (Fig. 1C). iKeap1 did not alter *Nrf2* mRNA expression (Fig. 1D). Results in Fig. 1E confirmed that nuclear Nrf2 protein levels were increased in iKeap1-treated murine osteoblasts, suggesting that stabilized Nrf2 protein translocated from cytosol to cell nuclei, the initial step for Nrf2 cascade activation [36].

Expression levels of Nrf2-ARE-dependent mRNAs, including *HO1*, *NQO1*, and *GCLC* (tested by qRT-PCR assays), were significantly increased in iKeap1-treated murine osteoblasts (Fig. 1F). Moreover, protein levels of HO1, NQO1, and GCLC were increased as well (Fig. 1G). These results implied that iKeap1 activated Nrf2 signaling cascade in primary murine osteoblasts. iKeap1 at 10 μM was more potent than at 2.5 μM in activating Nrf2 cascade in murine osteoblasts (Fig. 1A–G), showing a dose-dependent response.

In the primary human osteoblasts, Keap1-Nrf2 association was disrupted by iKeap1 (10 μM) treatment (Fig. 1H), which was accompanied by Nrf2 protein stabilization and cytosol accumulation (Fig. 1I). mRNA levels of Nrf2-ARE-dependent genes, including *HO1*, *NQO1*, and *GCLC*, were significantly elevated in iKeap1 (10 μM)-treated human osteoblasts (Fig. 1J). These results showed that iKeap1 activated Nrf2 cascade in murine and human osteoblasts.

### iKeap1 ameliorates H_2_O_2_-induced ROS production and oxidative injury in murine and human osteoblasts

**Fig. 2:**
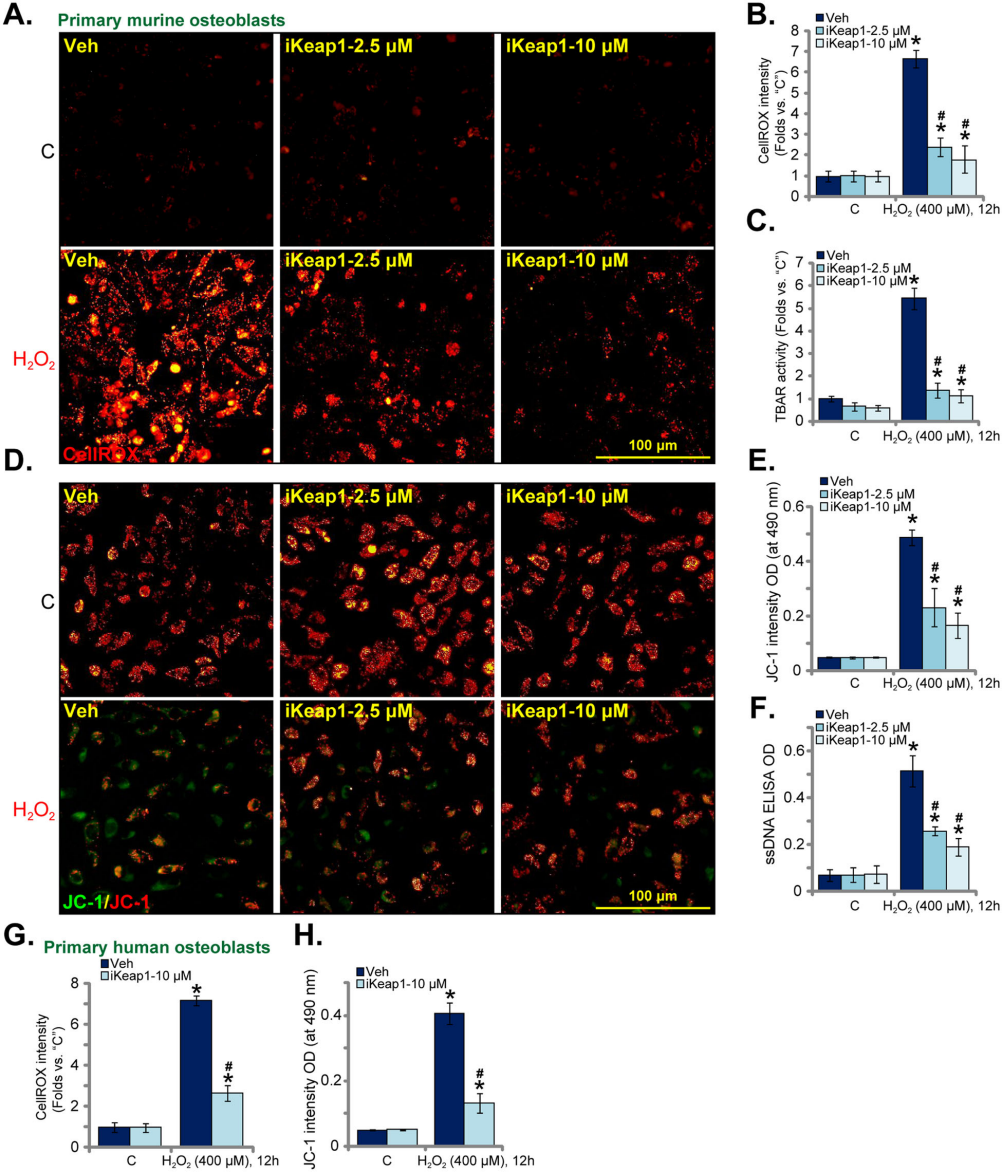
iKeap1 ameliorates H_2_O_2_-induced ROS production and oxidative injury in murine and human osteoblasts. The primary murine osteoblasts (**A**–**F**) or the primary human osteoblasts (**G** and **H**) were pretreated (for 2 h) with iKeap1 (2.5/10 μM), followed with or without H_2_O_2_ (400 μM) stimulation, and cells were cultured for applied time periods; ROS contents (CellROX intensity assay, **A**, **B**, **G**), lipid peroxidation (TBAR activity, **C**), mitochondrial depolarization (JC-1 staining assays, **D**, **E**, and **H**) and DNA damage [single strand DNA (ssDNA) ELISA OD, **F**] were tested by the mentioned assays, and results were quantified and normalized. Quantified values were mean ± standard deviation (SD, *n* = 5). “C” stands for the untreated control cells. *P < 0.05 vs. “C” cells. ^#^*P* < 0.05 vs. cells with H_2_O_2_ stimulation but “Veh” pretreatment. Experiments were repeated five times, with similar results obtained. Scale bar = 100 μm (**A** and **D**).

We have previously shown that activation of Nrf2 cascade can protect osteoblasts from H_2_O_2_ [4], we therefore analyzed whether iKeap1 could attenuate oxidative injury in osteoblasts. The primary murine osteoblasts were treated with H_2_O_2_ (400 μM, 6 h) and the cellular ROS contents (CellROX intensity) were significantly increased (Fig. 2A, B). Pretreatment with iKeap1 potently inhibited H_2_O_2_-induced ROS production in murine osteoblasts (Fig. 2A, B). Quantitative analyses showed that 10 μM of iKeap1 was more potent than 2.5 μM in suppressing H_2_O_2_-induced CellROX intensity increase (Fig. 2B). Single treatment of iKeap1 failed to alter cellular ROS contents in murine osteoblasts (Fig. 2A, B). To further support the anti-oxidant activity by the Keap1 inhibitor, we found that H_2_O_2_-induced lipid peroxidation was significantly inhibited after iKeap1 (2.5/10 μM) pretreatment (Fig. 2C). Lipid peroxidation in murine osteoblasts was quantified via TBAR activity assays (Fig. 2C).

With H_2_O_2_ stimulation mitochondrial membrane potential reduction, or mitochondrial depolarization, was detected (Fig. 2D, E). It was evidenced by accumulation of JC-1 green monomers (Fig. 2D, E). Importantly, mitochondrial depolarization in H_2_O_2_-treated murine osteoblasts was largely alleviated after iKeap1 (2.5/10 μM) pretreatment (Fig. 2D, E). Increased DNA damage was also detected in H_2_O_2_-treated murine osteoblasts, as the single-strand DNA (ssDNA) contents were increased (Fig. 2F). Pretreatment with iKeap1 (2.5/10 μM) potently alleviated DNA damage by H_2_O_2_ (Fig. 2F). iKeap1 again displayed a dose-dependent activity in suppressing lipid peroxidation, mitochondrial depolarization, and DNA damage, and being more effective at 10 μM (Fig. 2C–F).

In the primary human osteoblasts, H_2_O_2_ (400 μM) stimulation similarly induced ROS production (CellROX intensity increase, Fig. 2G) and mitochondrial depolarization (JC-1 green monomers accumulation, Fig. 2H). Such actions by H_2_O_2_ were largely inhibited by pretreatment with iKeap1 (10 μM) in human osteoblasts (Fig. 2G,H). Therefore iKeap1 significantly ameliorated H_2_O_2_-induced ROS production and oxidative injury in murine and human osteoblasts.

### iKeap1 ameliorates H_2_O_2_-induced death of murine and human osteoblasts

**Fig. 3:**
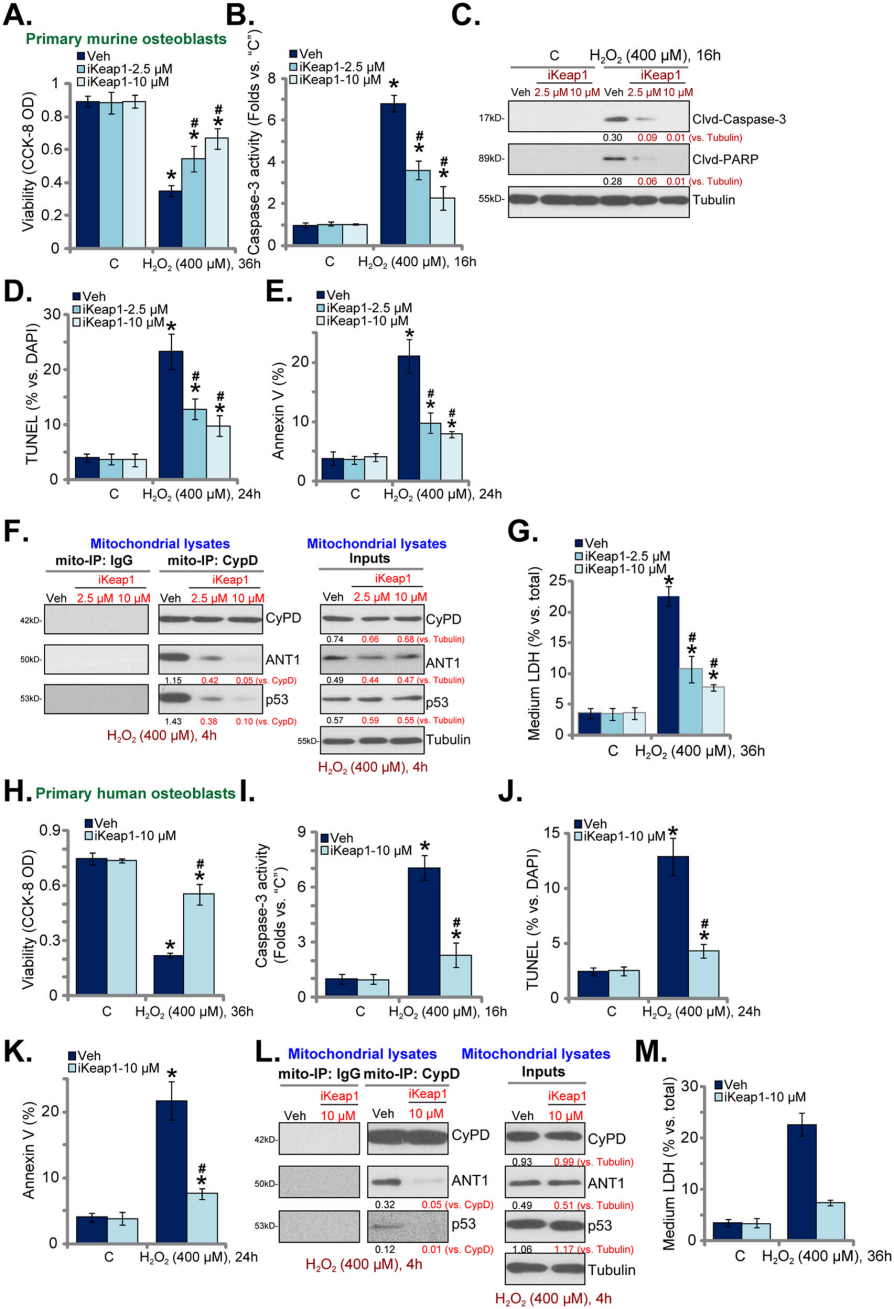
iKeap1 ameliorates H_2_O_2_-induced death of murine and human osteoblasts. The primary murine osteoblasts (**A**–**G**) or the primary human osteoblasts (**H**–**M**) were pretreated (for 2 h) with iKeap1 (2.5/10 μM), followed with or without H_2_O_2_ (400 μM) stimulation and osteoblasts were cultured for applied time periods; Cell viability was tested by CCK-8 assays (**A** and **H**); Caspase-3 activity was tested (**B** and **I**); Expression of apoptosis-associated proteins was tested by western blotting assays (**C**); Cell apoptosis was examined by nuclear TUNEL staining assays (**D** and **J**) and Annexin V FACS (**E** and **K**) assays, and results were quantified. Mitochondrial CyPD-ANT1-p53 association and their expressions were shown (**F** and **L**), and cell necrosis examined by quantifying medium LDH release (**G** and **M**). Expressions of the listed proteins were quantified and normalized to the loading control. Quantified values were mean ± standard deviation (SD, *n* = 5). “C” stands for the untreated control cells. *P < 0.05 vs. “C” cells. ^#^*P* < 0.05 vs. cells with H_2_O_2_ stimulation but “Veh” pretreatment. Experiments were repeated five times, with similar results obtained.

The potential effect of iKeap1 on H_2_O_2_-induced osteoblast cell death was studied next. In line with our previous findings [4], H_2_O_2_ (400 μM) stimulation led to dramatic viability (CCK-8 OD) reduction in primary murine osteoblasts (Fig. 3A), which was largely ameliorated by iKeap1 (2.5/10 μM) pretreatment (Fig. 3A). Furthermore, iKeap1 largely inhibited H_2_O_2_ (400 μM)-induced caspase-3 activation (Fig. 3B) and caspase-3-PARP cleavages (Fig. 3C). Nuclear TUNEL staining assays were performed to examine cell apoptosis. As shown, iKeap1 (2.5/10 μM) potently ameliorated H_2_O_2_-induced increase in TUNEL-positive nuclei ratio in murine osteoblasts (Fig. 3D). Moreover, the ratio of Annexin V-positive murine osteoblasts was significantly increased following H_2_O_2_ (400 μM) stimulation (Fig. 3E), which was inhibited by iKeap1 (2.5/10 μM) (Fig. 3E). Again,10 μM of iKeap1-induced anti-apoptosis activity was significant more potent than at 2.5 μM (Fig. 3B–E).

Different groups [7, 37–39], including ours [4], have reported that H_2_O_2_ and other oxidative stimuli can induce programmed necrosis in osteoblastic cells/osteoblasts. Following H_2_O_2_ stimulation p53 could translocate to mitochondria to form a complex with two mitochondrial permeability transition pore (mPTP) components: cyclophilin D (CyPD) and adenine nucleotide translocase-1 (ANT1) [40–42], causing mPTP opening and cell necrosis [40–42]. This process could be inhibited by Nrf2 cascade activation [4, 7, 33, 43]. We found that CyPD immunoprecipitated with ANT1 and p53 in the mitochondria of H_2_O_2_-treated primary murine osteoblasts (Fig. 3F), which was largely inhibited by iKeap1 pretreatment (Fig. 3F). Mitochondrial expressions of CyPD, ANT1, and p53 were however unchanged (Fig. 3F, “Inputs”). Significantly, H_2_O_2_-induced cell necrosis, tested by increased medium LDH release (Fig. 3G), was also attenuated by iKeap1. These results suggested that iKeap1 ameliorated H_2_O_2_-induced programmed necrosis in murine osteoblasts.

Similar results were obtained in the primary human osteoblasts, where iKeap1 (10 μM) pretreatment largely attenuated H_2_O_2_ (400 μM)-induced viability (CCK-8 OD) reduction (Fig. 3H). Furthermore, H_2_O_2_-induced apoptosis activation, evidenced by increases in caspase-3 activity (Fig. 3I), TUNEL-positive nuclei ratio (Fig. 3J) and Annexin V-positive cells (Fig. 3K), was alleviated following iKeap1 (10 μM) pretreatment. In the human osteoblasts, H_2_O_2_-induced CyPD-ANT1-p53 association (Fig. 3L) and medium LDH release (Fig. 3M) were largely inhibited by iKeap1 (10 μM), indicating the blockage of programmed necrosis cascade. Single treatment with iKeap1 failed to significantly affect cell viability, apoptosis, and programmed necrosis in murine osteoblasts (Fig. 3A–G) and human osteoblasts (Fig. 3H–M). Therefore iKeap1 potently ameliorated H_2_O_2_-induced apoptosis and programmed necrosis in murine and human osteoblasts.

### In murine and human osteoblasts iKeap1 ameliorates dexamethasone- and nicotine-induced oxidative injury and cell death

Studies have shown that DEX treatment can induce significant ROS production and oxidative injury, serving as a primary mechanism of osteoblast death [33, 43–47]. Conversely, activation of Nrf2 cascade could efficiently protect osteoblasts from DEX-induced oxidative injury [33, 43–47]. Similarly, nicotine from cigarette smoke is able to induce oxidative injury to osteoblasts, which is associated with pathogenesis of osteoporosis [48–51].

**Fig. 4:**
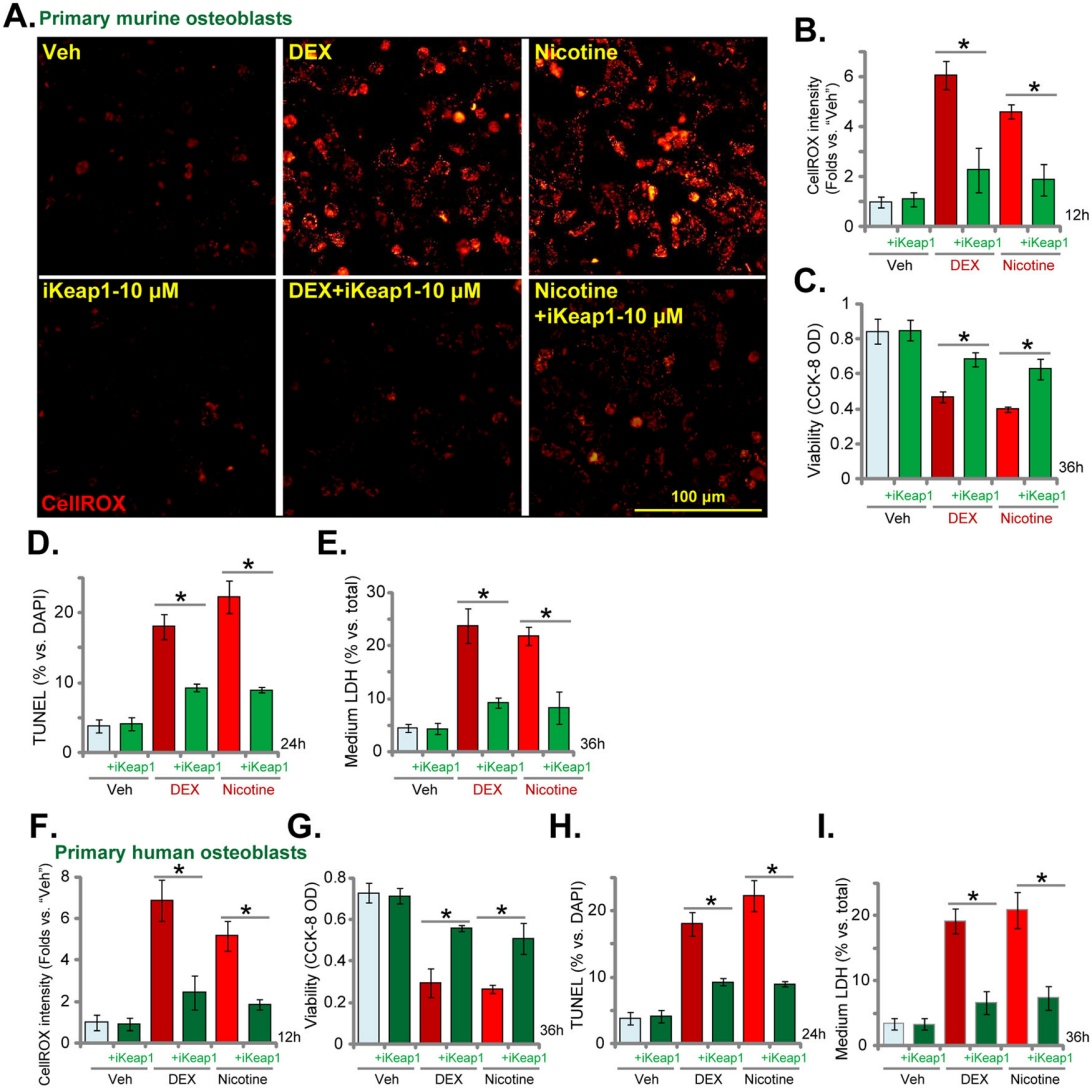
In murine and human osteoblasts iKeap1 ameliorates dexamethasone- and nicotine-induced oxidative injury and cell death. The primary murine osteoblasts (**A**–**F**) or the primary human osteoblasts (**G**–**J**) were pretreated (for 2 h) with iKeap1 (10 μM), followed with or without dexamethasone (DEX, 2 μM)/nicotine (1 μM) stimulation, cells were further cultured for applied time periods, ROS production, cell viability, apoptosis, and necrosis were tested by CellROX staining (**A**, **B** and **F**), CCK-8 (**C** and **G**), nuclear TUNEL staining (**D** and **H**), and medium LDH release (**E** and **I**) assays, respectively, and results were normalized and quantified. Quantified values were mean ± standard deviation (SD, *n* = 5). “Veh” stands for the vehicle control (0.1% DMSO). *P < 0.05. Experiments were repeated five times, with similar results obtained. Scale bar = 100 μm (**A**).

We next tested whether iKeap1 could protect osteoblasts from these two stimuli. The CellROX fluorescence images, Fig. 4A, indicated that ROS intensity was significantly increased in primary murine osteoblasts after DEX (2 μM) and nicotine (1 μM) stimulation, which was largely attenuated with iKeap1 (10 μM) pretreatment (Fig. 4A, B). DEX- and nicotine-induced viability (CCK-8 OD) reduction (Fig. 4C), apoptosis activation (TUNEL-positive nuclei ratio increase, Fig. 4D), and cell necrosis (medium LDH release, Fig. 4E) were largely attenuated by iKeap1 in primary murine osteoblasts. Similar results were obtained in the primary human osteoblasts, where iKeap1 inhibited DEX- and nicotine-induced ROS production (Fig. 4F), viability reduction (Fig. 4G), apoptosis induction (Fig. 4H), and cell necrosis (Fig. 4I).

### Nrf2 activation is absolutely required for iKeap1-induced osteoblast cytoprotection against H_2_O_2_

**Fig. 5:**
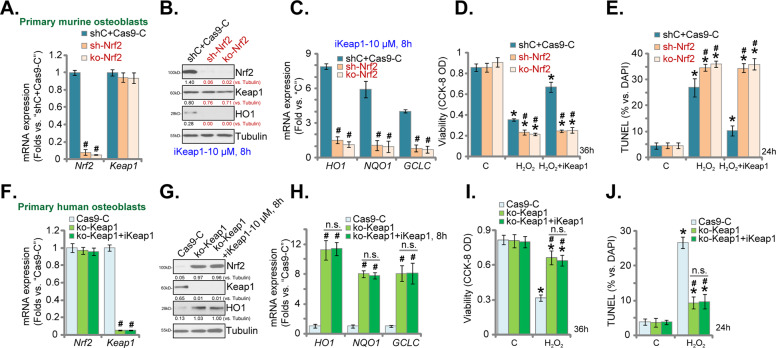
Nrf2 activation is absolutely required for iKeap1-induced osteoblast cytoprotection against H_2_O_2_. Stable murine osteoblasts expressing the Nrf2 shRNA (“sh-Nrf2”), a CRISPR/Cas9-Nrf2-KO-GFP-puro construct (“ko-Nrf2”) or scramble control shRNA plus the CRISPR/Cas9 empty vector (“shC+Cas9-C”) were established; *Keap1* and *Nrf2* mRNA expressions were tested by qRT-PCR assays (**A**); The osteoblasts were treated with iKeap1 (10 μM) and further cultured for indicated time periods, expressions of listed proteins (**B**) and mRNAs (**C**) were tested; Alternatively, the osteoblasts were pretreated with iKeap1 (10 μM) for 2 h, followed by H_2_O_2_ (400 μM) stimulation, cells were cultured for applied time periods; Cell viability and apoptosis were tested by CCK-8 (**D**) and TUNEL staining (**E**) assays, respectively. Stable murine osteoblasts expressing a CRISPR/Cas9-Keap1-KO-GFP-puro construct (“ko-Keap1”) or the CRISPR/Cas9 empty vector (“Cas9-C”) were established; *Keap1* and *Nrf2* mRNA expressions were tested by qRT-PCR assays (**F**); The ko-Keap1 osteoblasts were also treated with or without iKeap1 (10 μM) for applied time periods, expressions of listed proteins (**G**) and mRNAs (**H**) were tested; Alternatively, the ko-Keap1 osteoblasts were pretreated with iKeap1 (10 μM) s, followed by H_2_O_2_ (400 μM) stimulation and cells were cultured for applied time periods; Cell viability and apoptosis were tested by CCK-8 (**I**) and TUNEL staining (**J**) assays, respectively. Expressions of the listed proteins were quantified and normalized to the loading control. Quantified values were mean ± standard deviation (SD, *n* = 5). “C” stands for the untreated control cells. *P < 0.05 vs. “C” cells. ^#^*P* < 0.05 vs. “shC+Cas9-C”/“Cas9-C” osteoblasts. “n.s.” stands for the non-statistical difference (**H**–**J**). Experiments were repeated five times, with similar results obtained.

To test whether Nrf2 cascade activation is required for iKeap1-induced osteoblast cytoprotection against H_2_O_2_, we utilized genetic strategies to silence Nrf2. As reported early [4] primary murine osteoblasts were transfected with Nrf2 shRNA lentiviral particles and stable osteoblasts were established via selection by puromycin. These cells were named as “sh-Nrf2” osteoblasts. Alternatively, a CRISPR/Cas9-Nrf2-KO-GFP-puro construct (see our previous study [4]) was transduced to murine osteoblasts, and single stable “ko-Nrf2” osteoblasts were established by FACS-mediated sorting and Nrf2-KO screening. As compared to the control osteoblasts with scramble control shRNA plus the CRISPR/Cas9 empty vector (“shC+Cas9-C”), *Nrf2 mRNA* levels were almost depleted in sh-Nrf2 osteoblasts and ko-Nrf2 osteoblasts (Fig. 5A), where *Keap1* mRNA levels were unchanged (Fig. 5A).

In murine osteoblasts, iKeap1 (10 μM, 6 h)-induced Nrf2 protein stabilization (Fig. 5B) as well as mRNA (Fig. 5C) and protein (Fig. 5B) expressions of Nrf2-dependent genes were almost completely reversed after Nrf2 shRNA or KO. H_2_O_2_-induced viability (CCK-8 OD) reduction (Fig. 5D) and cell apoptosis (TUNEL-positive nuclei ratio increase, Fig. 5E) were intensified with Nrf2 depletion. Significantly, in Nrf2-silenced or Nrf2-KO murine osteoblasts, iKeap1 (10 μM) was completely ineffective against H_2_O_2_-induced cytotoxicity and apoptosis (Fig. 5D, E). These results supported that activation of Nrf2 signaling cascade is required for iKeap1-induced osteoblast cytoprotection against H_2_O_2_.

Next, a CRISPR/Cas9-Keap1-KO-GFP-puro construct was transduced to murine osteoblasts, single stable osteoblasts were again established (“ko-Keap1” osteoblasts). *Keap1* mRNA (Fig. 5F) and protein (Fig. 5G) were depleted in “ko-Keap1” osteoblasts, where *Nrf2* mRNA levels were unchanged (Fig. 5F). Keap1 KO induced Nrf2 protein stabilization as well as increased mRNA (Fig. 5H) and protein (Fig. 5G) expressions of Nrf2-dependent genes. The ko-Keap1 murine osteoblasts were protected from H_2_O_2_-induced viability reduction (Fig. 5I) and apoptosis (Fig. 5J). Importantly, Nrf2 was constitutively active in the ko-Keap1 murine osteoblasts, and treatment with iKeap1 (10 μM) failed to further increase Nrf2 activation (Fig. 5G, H). In addition, the Keap1 inhibitor was unable to offer further osteoblast cytoprotection against H_2_O_2_ (Fig. 5I, J). Nrf2 or Keap1 silencing/KO, by itself, did not affect viability (Fig. 5D, I) and apoptosis (Fig. 5E, J) in murine osteoblasts. These results further supported that activation of Nrf2 cascade is absolutely required for iKeap1-induced osteoblast cytoprotection against H_2_O_2_.

## Discussion

Studies have shown that Nrf2 activation using various pharmacological agents could protect osteoblasts/osteoblastic cells from H_2_O_2_-induced oxidative injury and cell death [7, 9, 10]. However, most of these agents are not direct Nrf2 activators and are often utilized at relatively higher concentrations [4, 10, 52–54]. A very recent study has identified iKeap1 as a novel and highly efficient Nrf2 activator [24]. iKeap1 is engaged in the Nrf2-binding pocket of Keap1 and locates at the entrance to the tunnel formed by the β-barrel [24]. Nuclear magnetic resonance analyses confirmed a direct binding between iKeap1 and Keap1 [24]. Surface plasmon resonance (SPR) studies demonstrate that iKeap1 binds to Keap1 at a binding affinity of 114 nM [24]. A fluorescence polarization assay results found that iKeap1 can displace Nrf2 peptide at the IC_50_ of 258 nM [24]. Its potential effect on H_2_O_2_-induced osteoblast injury was studied here.

In human and murine osteoblasts, iKeap1 activated Nrf2 cascade signaling, causing Keap1-Nrf2 disassociation, Nrf2 protein stabilization, cytosol accumulation, and nuclear translocation. In addition, the novel Keap1 inhibitor increased ARE activity and expression of Nrf2-dependent genes (*HQ1*, *NQO1*, and *GCLC*) in murine and human osteoblasts. Functional studies showed that iKeap1 largely attenuated H_2_O_2_-induced ROS accumulation, lipid peroxidation, mitochondrial depolarization, and DNA breaks in murine and human osteoblasts. Furthermore, H_2_O_2_-induced osteoblast apoptosis was significantly ameliorated after iKeap1 pretreatment.

Besides apoptosis, H_2_O_2_ could promote p53 translocation to mitochondria to form the CyPD-ANT1-p53 complex, leading to mPTP opening and cell necrosis [4, 41, 42]. Inhibition of this process can exert significant osteoblastic cytoprotection [4, 41, 42]. Here we found that H_2_O_2_-induced CyPD-ANT1-p53 association and necrosis were largely inhibited by iKeap1. These results further explained the superior osteoblast cytoprotective activity by the novel Keap1 inhibitor.

DEX-induced osteoblast cell death is an important cause of osteoporosis and osteonecrosis [48]. DEX could induce significant ROS production and oxidative injury in osteoblasts/osteoblastic cells, serving as a key mechanism of osteoblast cell death [33, 43–47]. Activation of Nrf2 cascade, using genetic methods or pharmacological strategies, could exert significant osteoblast cytoprotection and inhibit DEX-induced cytotoxicity. Liang et al. showed that phosphoglycerate kinase 1 shRNA or KO induced Keap1 methylglyoxal (MG) modification to activate Nrf2 cascade, protecting osteoblasts from DEX-induced oxidative injury and apoptosis [33]. Zhao et al. reported that Keap1 silencing by microRNA-200a activated Nrf2 signaling cascade and inhibited DEX-induced ROS production and osteoblast cell death [46]. Zhuang et al. found that microRNA-107 inhibition upregulated CAB39 (calcium-binding protein 39) to activate AMP-activated protein kinase-Nrf2 signaling cascade, thereby protecting osteoblasts from DEX-induced oxidative injury and cytotoxicity [44]. Moreover, three different agents, fibroblast growth factor 23 (FGF23), SC79 (an Akt activator), and Icariside II, can activate Akt-dependent Nrf2 cascade and inhibited DEX-induced oxidative injury in osteoblasts [43, 45, 47]. Here we found that iKeap1 inhibited DEX-induced ROS production and osteoblast cell apoptosis. Therefore iKeap1 could be a novel strategy targeting DEX-induced osteoblast injury and osteoporosis.

Existing studies have reported that nicotine exposure could inhibit proliferation, differentiation, alkaline phosphatase activity, oxidative metabolism, and collagen synthesis as well as calcium absorption and mineralized nodule formation in osteoblastic cells and osteoblasts [49–51]. Furthermore, nicotine is shown to induce human osteoblast apoptosis, which could also be linked to cigarette smoke-induced osteoporosis and dental implant failure [49–51]. Marinucci et al. found that nicotine-induced osteoblast cell apoptosis was driven by H_2_O_2_-induced glyoxalase 1 inhibition and MG-H1 accumulation [49]. Liang et al. reported that nicotine altered genes and signaling pathways associated with bone formation in the rat osteoblasts, and induced osteoblast cell apoptosis [50]. Ma et al. reported that nicotine decreased expression of osteogenic and angiogenic genes, including TGFβ, BMP-2, PDGF-AA, and VEGF, in primary rat osteoblasts [51]. Here we found that nicotine-induced oxidative injury and apoptosis in murine and human osteoblasts were potently alleviated after iKeap1 pretreatment. Therefore, this novel Keap1 inhibitor might have important translational value for the treatment of nicotine-associated osteoblast injury.

Our results supported that Nrf2 cascade activation is absolutely required for iKeap1-induced cytoprotective actions in H_2_O_2_-treated osteoblasts. In murine osteoblasts, Nrf2 shRNA or CRISPR/Cas9-induced Nrf2-KO intensified H_2_O_2_-induced oxidative stress and cell apoptosis. Significantly, iKeap1-induced osteoblast cytoprotection against H_2_O_2_ was completely reversed with Nrf2 silencing or KO. Conversely, Keap1 KO in murine osteoblasts induced robust Nrf2 activation and mimicked iKeap1-induced actions. Importantly, iKeap1 failed to offer further cytoprotection against H_2_O_2_ in Keap1-KO murine osteoblasts. Thus, iKeap1-induced osteoblast cytoprotection relies on activation of Keap1-Nrf2 cascade.

## Conclusion

Taken together, iKeap1 activated Nrf2 signaling cascade to inhibit H_2_O_2_-induced oxidative injury and osteoblast death. This novel Nrf2 activator could be a novel strategy to protect osteoblasts from various oxidative stimuli.
